# Supplementary material for: A Predicted Helix-Turn-Helix Core Is Critical for Bacteriophage Kil Peptide to Disrupt Escherichia coli Cell Division
Source: Antibiotics (Basel). 2025 Jan 8;14(1):52. doi: 10.3390/antibiotics14010052 (PMC11762379; doi:10.3390/antibiotics14010052)
Supplement: Supplementary file 1 [file antibiotics-14-00052-s001.zip › antibiotics-3395094-supplementary.pdf]

SUPPLEMENTARY MATERIALS

A predicted helix-turn-helix core is critical for bacteriophage Kil peptide to disrupt *Escherichia coli* cell division

Arindam Naha[1#], Todd A. Cameron[1] and William Margolin[1]

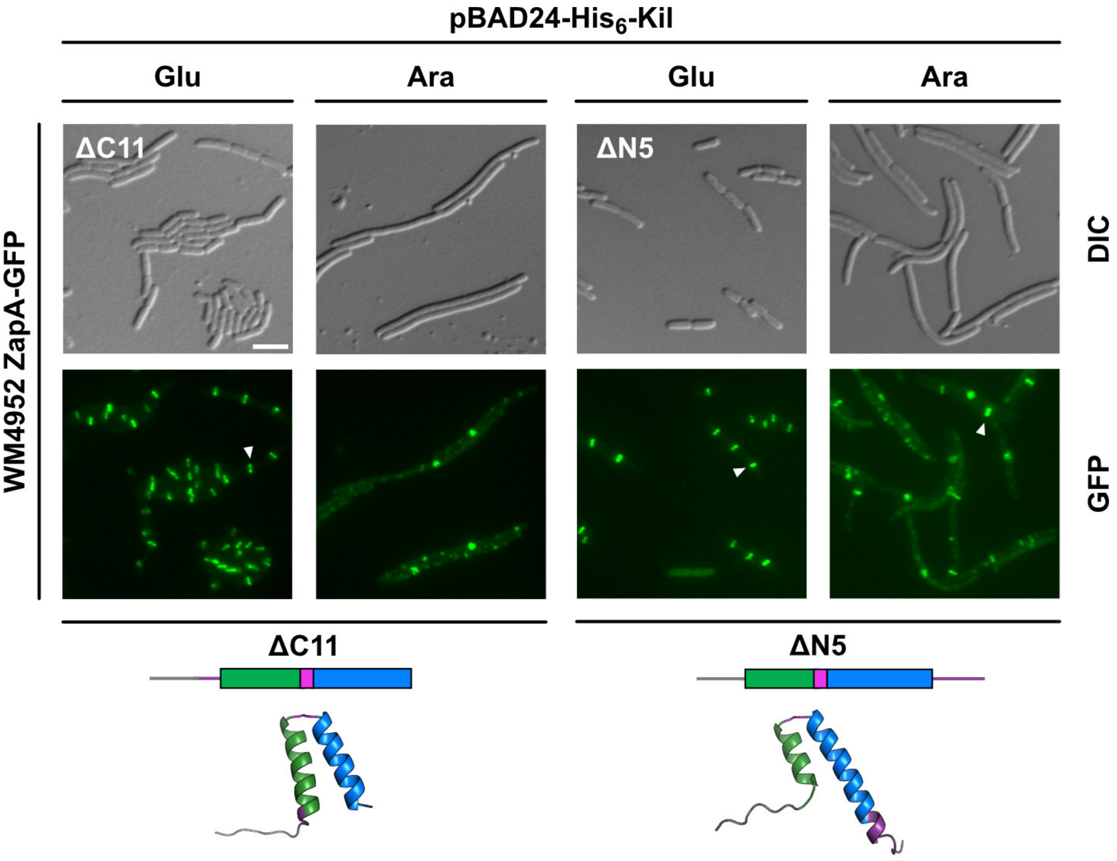

Figure S1. Shorter λ Kil truncations also inhibit *E. coli* cytokinesis. (A) Fluorescence microscopic images of WM4952 expressing ZapA-GFP at its native chromosomal locus along with His-tagged KilΔN5 or ΔC11 truncations from pBAD24 under repressing (0.2% glucose, Glu) or inducing (0.2% arabinose, Ara) conditions. Arrowheads indicate intact Z-rings. Schematic diagrams of the Kil derivatives are presented at the bottom of each corresponding micrograph, along with AlphaFold 3-modeled structures. Scale bar, 5 μm.

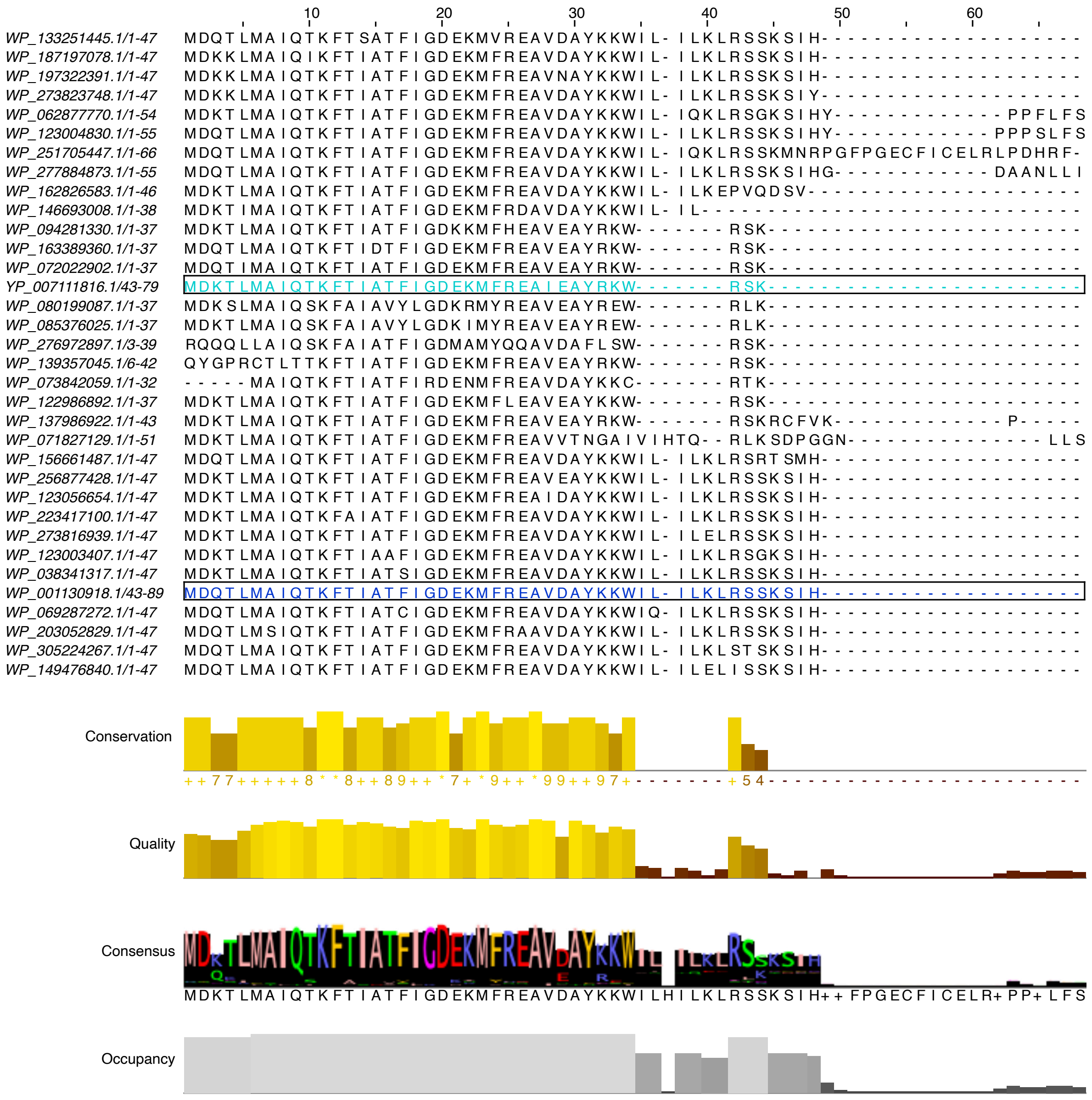

Figure S2. Multiple sequence alignment of λ Kil-like protein sequences. MAFFT E-INS-i alignment of λ Kil-like sequences obtained from a BLAST search of the RefSeq database using the λ Kil sequence as the query. Sequences with 98% or greater redundancy were removed. The sequences corresponding to λ Kil and HK629 Kil are highlighted in blue and teal, respectively. The sequence alignment begins at the experimentally verified translational start site of λ Kil. Multiple sequence alignment was visualized using Jalview.
